# Developing renal allograft surveillance strategies – urinary biomarkers of cellular rejection

**DOI:** 10.1186/s40697-015-0061-x

**Published:** 2015-08-18

**Authors:** Patricia Hirt-Minkowski, Sacha A De Serres, Julie Ho

**Affiliations:** Clinic for Transplant Immunology and Nephrology, University Hospital Basel, Petersgraben 4, 4031 Basel, Switzerland; Transplantation Unit, Renal Division, Department of Medicine, CHU de Québec – L’Hôtel-Dieu, Faculty of Medicine, Laval University, 11 Côte du Palais, Québec, QC G1R 2J6 Canada; Internal Medicine & Immunology, Sections of Nephrology & Biomedical Proteomics, University of Manitoba, GE421C Health Sciences Centre, 820 Sherbrook Street, Winnipeg, MB R3A 1R9 Canada; Manitoba Centre for Proteomics and Systems Biology, Health Sciences Centre, Winnipeg, MB Canada; Department of Immunology, University of Manitoba, Winnipeg, MB Canada

**Keywords:** Non-invasive monitoring, Post-transplant surveillance, Urinary biomarker, CXCR3 chemokines, CXCL9, CXCL10

## Abstract

**Purpose of review:**

Developing tailored immunosuppression regimens requires sensitive, non-invasive tools for serial post-transplant surveillance as the current clinical standards with serum creatinine and proteinuria are ineffective at detecting subclinical rejection. The purpose of this review is: (i) to illustrate the rationale for allograft immune monitoring, (ii) to discuss key steps to bring a biomarker from bench-to-bedside, and (iii) to present an overview of promising biomarkers for cellular rejection.

**Sources of information:**

PubMed.

**Findings:**

Recent multicentre prospective observational cohort studies have significantly advanced biomarker development by allowing for the adequately powered evaluation of multiple biomarkers capable of detecting allograft rejection. These studies demonstrate that urinary CXCR3 chemokines (i.e. CXCL9 and CXCL10) are amongst the most promising for detecting subclinical inflammation; increasing up to 30 days prior to biopsy-proven acute rejection; decreasing in response to anti-rejection therapy; and having prognostic significance for the subsequent development of allograft dysfunction. Urinary CXCR3 chemokines are measured by simple and cost-effective ELISA methodology, which can readily be implemented in clinical labs.

**Limitations:**

Many biomarker studies are performed in highly selected patient groups and lack surveillance biopsies to accurately classify healthy transplants. Few validation studies have been done in unselected, consecutive patient populations to characterize population-based diagnostic performance.

**Implications:**

Based on these data, prospective interventional trials should be undertaken to determine if chemokine-based post-transplant monitoring strategies can improve long-term renal allograft outcomes. This last step will be necessary to move novel biomarkers from the bench-to-bedside.

## Abrégé

**Objet de l’étude:**

L’élaboration sur mesure d’un schéma posologique d’immunosuppression requiert des outils adaptés et non invasifs de contrôle systématique à la suite de la greffe, étant donné l’inefficacité des normes cliniques actuelles en ce qui a trait à la créatininémie et à la protéinurie dans la détection des rejets subcliniques. L’objet de cette étude sont les suivants: (i) illustrer la raison d’être d’un contrôle de la réponse immunitaire à la greffe allogénique, (ii) déterminer les étapes clés visant à transposer un biomarqueur du laboratoire au chevet du patient, et (iii) présenter un aperçu des biomarqueurs prometteurs dans le rejet cellulaire.

**Conclusion:**

Les études prospectives, multicentriques et comparatives récentes ont fait considérablement progresser l’élaboration de biomarqueurs en permettant l’évaluation adéquate de nombre d’entre eux, capables de détecter le rejet de greffe allogénique. Ces études démontrent que les chimiokines urinaires CXCR3 (c.-à-d. CXCL9 et CXCL10) sont parmi les biomarqueurs les plus prometteurs dans la détection d’inflammation subclinique : elles augmentent jusqu’à 30 jours précédant un rejet aigu prouvé par biopsie, leur nombre diminue en réponse à la thérapie antirejet et elles ont des qualités pronostiques importantes dans l’apparition subséquente de dysfonctions. Les chimiokines urinaires CXCR3 se mesurent grâce à la méthodologie ELISA, simple et efficiente, et dont la mise en œuvre peut se faire sans tarder dans les laboratoires cliniques.

**Limites de l’étude:**

Plusieurs études sur les biomarqueurs ont pour sujets des groupes de patients soigneusement choisis, et sont exemptes de biopsies de surveillance qui permettraient de classifier avec justesse les greffes saines. Peu d’études de validation ont été effectuées sur des échantillons de patients non sélectionnés et consécutifs dans le but de représenter la classification du rendement au sein de la population.

**Répercussions:**

À la lumière de ces données, il importerait d’entreprendre des essais prospectifs d’intervention afin de déterminer si les stratégies de contrôle suivant la greffe et basées sur les chimiokines sont susceptibles d’améliorer les résultats de la greffe rénale allogénique à long terme. Cette dernière étape sera nécessaire afin de transposer les nouveaux biomarqueurs du laboratoire au chevet du patient.

## What was known before:

Routine post-transplant surveillance with serum creatinine and proteinuria are not sensitive enough to detect subclinical rejection, which can impact long-term renal allograft outcomes. Urinary biomarkers may provide a sensitive, non-invasive means for serial monitoring of renal allografts.

## What this adds:

This review provides an overview of the rationale and requirements for biomarker development, which are broadly applicable, and discusses the current state of the literature for urinary biomarkers of cellular rejection, and the future of biomarker research.

## Introduction

Transplantation is the therapy of choice for many patients with end organ failure. One of the major challenges of transplantation is the optimization of immunosuppressive therapy to balance the risk of rejection from under-immunosuppression against the risk of infection and malignancy resulting from over-immunosuppression [1, 2]. The ideal regimen would provide the minimum immunosuppression for each individual patient that is required to prevent subclinical and clinical rejection while limiting infections that have a negative impact on graft survival (e.g. polyoma virus). Developing tailored immunosuppression regimens requires sensitive, non-invasive tools for serial monitoring following drug minimization/withdrawal protocols to detect subclinical inflammation prior to injury, and also to follow the response to anti-rejection treatment. Indeed, novel post-transplant monitoring tools may help develop personalized medical care to improve graft survival. The purpose of this review is to provide the rationale for immune monitoring of renal allografts and to discuss key steps to bring a biomarker from bench-to-bedside. Finally, we will provide an overview of promising biomarkers for cellular rejection that have demonstrated potential to be translated into clinical practice.

### Why are non-invasive monitoring tools needed?

It has previously been common to state that although short-term renal allograft survival has improved as a result of improved immunosuppression and decreased rates of acute rejection, long-term graft survival has not changed [3, 4]. However recent reports suggest light at the end of the tunnel. Long-term renal allograft histology [5] and graft survival [6, 7] have improved for the recipients of both living and deceased donor kidneys. These gains are encouraging and demonstrate the potential for improving clinical outcomes; nevertheless graft loss remains a clinically evident problem. Indeed, the return to dialysis following graft loss is associated with a three-fold increased risk of death, immunological sensitization that may impede re-transplantation, a lower quality of life, and increased costs [8, 9]. United States Renal Data System (USRDS) studies demonstrate that adjusted patient survival after graft loss is less than 40 % at ten years compared to greater than 75 % ten-year survival with a functioning renal transplant [9]. Similarly, Canadian Organ Replacement Registry (CORR) data also demonstrates that graft loss is an independent predictor of mortality, with a three-fold increased risk of death compared to patients who maintain graft function [8].

Several groups have evaluated the causes and histopathologic lesions associated with late graft failure and consistently found that the underlying causes of graft loss are largely identifiable, primarily immune-mediated, and therefore potentially amenable to intervention [10–13]. In a cohort of 315 consecutive patients, late graft loss was commonly associated with chronic antibody-mediated rejection (AMR) (55 %); recurrent/de novo autoimmune glomerular disease (18 %); interstitial fibrosis and tubular atrophy (IFTA) alone (9 %); IFTA associated with polyoma virus nephropathy (5 %) or acute cellular rejection (14 %) [10]. As alloimmune-mediated injury remains the most common mechanism leading to graft failure it appears that under-immunosuppression remains a dominant long-term problem.

Surveillance biopsies between 4 to 12 months post-transplant in stable functioning grafts have shown that subclinical cellular rejection is a major acute rejection phenotype within the first year post-transplant and also an early predictor of subsequent graft failure [14–20]. The pathogenic potential of early (i.e. between 0-6 months) subclinical cellular rejection is supported by the fact that treatment of subclinical rejection in patients on cyclosporine-based therapy in two randomized, controlled trials leads to diminished histological injury and improved functional outcomes [21, 22]. Furthermore, effective treatment of subclinical cellular rejection in patients on modern immunosuppression resulted in similar long-term graft survival as patients without rejection [19]. Importantly, subclinical and clinical cellular rejection has been linked with the subsequent development of de novo donor specific antibody (DSA), with its associated risk of chronic AMR and graft loss [10, 19]. Taken together, these data suggest that subclinical cellular rejection is clinically significant and that effective therapy is available that can improve long-term renal allograft outcomes. Therefore, a key goal of post-transplant monitoring should be the early detection of alloimmune inflammation causing graft injury.

### Why is biomarker research important?

**Fig. 1:**
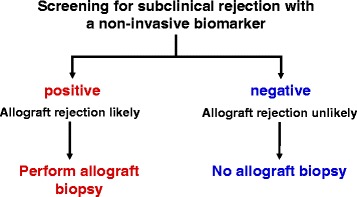
Non-invasive screening for subclinical rejection. Ideally, when a non-invasive biomarker is negative, we can exclude an inflammatory process with high confidence and a surveillance biopsy can be safely omitted. Alternatively, if the non-invasive screening biomarker is positive, an allograft biopsy can be performed to confirm rejection

Currently, we do not have a useful marker for subclinical rejection apart from surveillance biopsies. Thus, current strategies for monitoring the allograft remain limited, as serum creatinine cannot detect subclinical inflammatory processes. While surveillance biopsies remain the gold standard for diagnosis of subclinical rejection they are costly, associated with a small risk of complications, and subject to sampling error. Due to its invasive nature, surveillance biopsies are also limited for frequent serial monitoring. Therefore, novel non-invasive biomarkers should be capable of detecting subclinical inflammation and clearly out-perform serum creatinine [23]. Figure 1 summarizes the concept of screening for subclinical rejection with a non-invasive biomarker.

### What is required for biomarker development?

Broadly speaking, novel biomarker development has been categorized into the following phases: discovery, performance evaluation and impact determination [24]. While many biomarker discovery studies exist in highly selected patient groups, only a few validation studies have been done in unselected, consecutive patient populations to determine their true population-based diagnostic performance [25, 26]. Notably, none of the proposed non-invasive biomarkers for kidney allograft rejection has been evaluated in prospective interventional studies or been translated to routine clinical practice. In general, the stages to bring a biomarker from bench-to-bedside would ideally require:

#### Discovery

The biomarker discovery phase is characterized by identification of novel markers that correlate with rejection, utilizing either biased or unbiased approaches. These studies should be sufficiently powered, especially if multiple biomarkers are being assessed. Other inflammatory states (e.g. BKV, CMV, GN, UTI) are typically evaluated to determine the biomarker’s specificity for alloimmune inflammation. Finally, it is important that histology is available on all patients to prevent misclassification of cases. For example, controls should have concomitant normal histology to prevent misclassification of subclinical rejection.

#### Performance evaluation

Biomarkers need to be highly sensitive and specific, and this performance should be independently validated in separate cohorts. Large prospective, unselected consecutive cohorts are required to characterize true population-based diagnostic performance, and this has been performed by few transplant biomarker studies to date [25, 26]. Finally, in order for novel biomarkers to be translated from bench-to-bedside, it should be measurable on high throughput, inexpensive, robust and reproducible assays, with accessible lab equipment that would be available in clinical laboratories and follow Good Laboratory Practice Guidelines.

#### Impact determination

For novel biomarkers to have clinical utility, it is necessary to demonstrate that its diagnostic performance exceeds current clinical monitoring standards, by detecting inflammation/injury prior to graft dysfunction. For example, novel biomarkers which correlate with clinical rejection on indication biopsies may be hypothesis-generating but do not provide additional information beyond serum creatinine/proteinuria. Conversely, biomarkers which detect subclinical inflammation outperform serum creatinine/proteinuria by definition. Another important measure of clinical utility is the ability of a biomarker to correlate with response to therapy. Finally, to definitively characterize biomarker clinical utility it will be necessary to demonstrate that monitoring improves long-term renal allograft outcomes in a prospective, interventional trial. While some biomarkers meet all the preceding criteria for performance and clinical utility, there are no biomarkers that have been prospectively evaluated to determine their impact on allograft outcomes.

## Review

### Biomarkers for cellular rejection

Multiple novel biomarkers have been evaluated for the non-invasive detection of cellular rejection, however most have not been evaluated for subclinical rejection (e.g. FOXP3, Tim-3, fractalkine) or are not elevated in subclinical rejection (e.g. granzyme B, serpin B9, CXCL11) [reviewed in detail, 23]. Indeed only urinary CXCL9, CXCL10, granzyme A mRNA and perforin mRNA have been demonstrated to increase prior to biopsy-proven acute clinical rejection (i.e. rejection detected by indication biopsies performed due to functional decline) and to detect subclinical inflammation (i.e. inflammation detected by surveillance biopsies) [25–29]. For the purposes of this review, we will strictly focus on biomarkers that have passed the discovery phase with some validation and evidence of impact determination, defined as the ability to detect subclinical rejection or increase prior to an episode of acute clinical rejection, as these biomarkers have the greatest potential for translation to clinical practice.

### Urinary-cell mRNA

Cytotoxic T lymphocytes (CTL) can cause cell death through various mechanisms. Specifically, CTL can release perforin, which perforates cell membranes, causing direct cell death during rejection [30]. CTL also release granzymes A and B, which cause cell death via caspase-dependent and independent apoptosis [31]. In a biomarker discovery study with a highly selected patient population, Li et al. determined that urinary perforin and granzyme B mRNA were significantly elevated in patients with acute clinical rejection [32], and these observations were subsequently replicated in independent cohorts [33, 34]. These findings were extended by van Ham et al. who showed that urinary granzyme A mRNA and perforin mRNA were both associated with subclinical inflammation, however these findings have yet to be confirmed in an independent cohort [29].

The Clinical Trials in Organ Transplantation (CTOT) is a series of multicentre NIH-sponsored clinical studies with an overarching objective to improve long-term renal allograft outcomes. In the CTOT-04 study, Suthanthiran and colleagues evaluated urinary-cell mRNA as potential non-invasive diagnostic markers for acute cellular rejection in a large prospective cohort of 485 patients [35]. Of the 83 % urinary samples with sufficient RNA to pass quality control, they evaluated CD3ε, perforin, granzyme B, proteinase inhibitor 9, CD103, CXCL10, CXCR3, TGF-β mRNA and 18S rRNA [35]. They demonstrated that urinary CD3ε, CXCL10, perforin and granzyme B mRNA were all elevated in acute cellular rejection [35]. They subsequently developed a three-gene signature utilizing CD3ε mRNA, CXCL10 mRNA and18s rRNA (AUC 0.85) for detecting acute rejection and externally validated it in the CTOT-01 cohort [26, 35].

While the three-gene signature increased prior to biopsy-proven acute rejection, it is unclear if it detects subclinical rejection as only 5/43 analyzable cases were available [35]. Notably, only the Banff grade ≥ IA acute rejection (n = 43) and no rejection (n = 163) groups were used to calculate the diagnostic performance (AUC 0.85) [35]. Since the entire evaluable population (n = 244) was not included, this may be an artificially inflated AUC and conclusions cannot be made regarding its population-based diagnostic performance [36]. Indeed, the important question is whether or not any alloimmune or autoimmune inflammation is present and not just Banff ≥ IA rejection. Interestingly, the CTOT-04 and CTOT-01 studies evaluated urinary granzyme B mRNA, even though van Ham et al. demonstrated that it was not elevated in subclinical rejection, whereas granzyme A mRNA was elevated [29]. Finally, the limitations of extracting sufficient quality urinary mRNA for analysis (only 83% passed quality control in CTOT-04) is an inherent technical limitation to translating this assay from bench-to-bedside.

### Urinary CXCR3 chemokines

CXCR3 is a chemokine receptor that is expressed by activated T cells and natural killer cells and binds to CXCL9, CXCL10 and CXCL11 [37]. The chemokines CXCL9 and CXCL10 can be secreted by infiltrating inflammatory cells, renal tubular and mesangial cells; they are also involved in leukocyte recruitment and mediating the CD4 Th1 response (e.g. up-regulation of pro-inflammatory cytokine production like IFN-gamma, IL-2 and TNF-alpha) [38, 39]. In acute allograft rejection, CXCL9 and CXCL10 are highly expressed in infiltrating leukocytes and renal tubules, while CXCL9 expression is increased in the glomerulus [40, 41].

#### Urinary CXCL9

Urinary CXCL9 is significantly elevated in acute rejection [26, 27, 42–47]. Notably, urinary CXCL9 rises prior to an episode of biopsy-proven acute clinical rejection [26, 44] and decreases in response to therapy [26, 43, 44, 46]. Furthermore, urinary CXCL9 distinguishes subclinical tubulitis from normal and borderline histology (AUC 0.78), although these results have not yet been independently validated [27].

CTOT-01 was a multicentre prospective observational study of 280 adult and pediatric renal transplant recipients [26]. It was performed to validate multiple novel biomarkers using different methodologies: qPCR on urine sediment mRNA for CCR1, CCR5, CXCR3, CCL5, CXCL9, CXCL10, IL8, perforin and granzyme B; urine protein ELISA for CXCL9 and CXCL10; and SELDI TOF-MS for cleaved β2-microglobulin [26]. Of all these biomarkers, only urinary CXCL9 and CXCL10 protein, as well as CXCL9 mRNA and granzyme B mRNA, were significant univariate predictors of clinical Banff ≥ IA rejection [26]. CXCL9 was the best discriminator for acute rejection, and inclusion of CXCL9 mRNA and granzyme B mRNA in a multivariate model with CXCL9 protein did not improve its overall performance [26]. Notably, CXCL9 diagnosed acute rejection with an AUC 0.86, rose up to 30 days prior to clinical rejection and had a strong negative predictive value (NPV) 0.92 [26]. Importantly, CTOT-01 also demonstrated that urinary CXCL9 has prognostic significance, with elevated levels at 6 months being associated with a subsequent decline in eGFR at 24-months [26]. Finally, urinary CXCL9 protein correlated with subclinical Banff “i” and “t” scores; however, insufficient cases of subclinical rejection (n = 8) were available to validate its diagnostic performance [26].

CTOT-01 demonstrated that urinary CXCL9 had a similar diagnostic performance to CXCL10 (AUC 0.768, sensitivity 0.74, specificity 0.86, NPV 0.875) for detecting acute rejection, but they did not evaluate if these C-statistics were significantly different [26]. These findings are similar to those of Schaub et al. who demonstrated that urinary CXCL9 (AUC 0.78) and CXCL10 (AUC 0.79) had virtually identical diagnostic performances for the detection of subclinical tubulitis [27]. Finally, the combination of urinary CXCL9 and CXCL10 did not improve on the overall diagnostic performance [26], likely since both chemokines activate the same CXCR3 receptor and track the same pathophysiological pathway. The diagnostic performances of urinary CXCR3 chemokine proteins in different studies are summarized in Table 1.

**Table 1:** Diagnostic performance of urinary CXCR3 chemokine proteins

Biomarker				Acute cellular rejection ^g^					Subclinical rejection		
CXCL9	Population	Study Design	n ^a, b^	AUC	Sensitivity	Specificity	Ref	AUC	Sensitivity	Specificity	Ref
	Multi-center ^c^	Prospective, observational cohort	280	0.86	0.87	0.82	26	*Subclinical group too small for AUC*			
	Single center	Prospective, observational cohort	69	NR	0.93	0.89	44	*Subclinical group too small for AUC* ^e^			
	Single center	Case control	125	0.87	0.86	0.80	45	*Subclinical group too small for AUC*			
	Two centers	Case control	88					0.78	0.86	0.64	27
	Single center	Case control	113	0.92	0.85	0.90	42				
	Single center	Case control	99	NR	0.80	0.94	43				
	Single center	Case control	201	0.90	0.84	0.83	46				
	Single center ^f^	Case control	213	0.91	0.90	0.84	47				
CXCL10	Population	Study Design	n ^a, b^	AUC	Sensitivity	Specificity	Ref	AUC	Sensitivity	Specificity	Ref
	Multi-center ^c^	Prospective, observational cohort	280	0.77	0.74	0.86	26	*Subclinical group too small for AUC*			
	Single center	Prospective, observational cohort	213	0.74	0.63	0.80	25	0.69	0.61	0.72	25
	Two centers	Case control	88					0.79	0.68	0.90	27
	Single center	Case control	91	0.84	0.78	0.59	28	0.85	0.73	0.73	28
	Single center ^d^	Case control	51	0.88	0.77	0.60	52	0.81	0.59	0.67	52
	Single center	Case control	54					0.77 ^e^	0.62	0.95	48
	Single center ^c^	Case control	125	0.83	0.80	0.76	45	*Subclinical group too small for AUC*			
	Single center	Case control	113	0.93	0.89	0.81	42				
	Single center	Case control	99	NR	0.86	0.91	43				
	Single center	Case control	201	0.81	0.65	0.97	46				
	Single center ^f^	Case control	213	0.80	0.70	0.76	47				

^a^ Numbers of patients (not urine numbers), some studies are confounded with repeated measures; ^b^ Numbers in the AUC analysis, not the overall study population; ^c^ Pediatric population included; ^d^ Pediatric population only; ^e^ Subclinical refers to the AUC 4-5 days prior to clinical rejection for these studies; ^f^ Rabant et al. performed a prospective observational study of patients with indication biopsies – the data reported here are for the TCMR sub-group analysis; ^g^ Rejection is scored by the Banff criteria, but the definitions used vary by study. *NR* not reported

#### Urinary CXCL10

There is a significant body of evidence demonstrating that urinary CXCL10 is a sensitive marker for inflammation, and a number of groups have shown that urinary CXCL10 is associated with acute rejection [25–28, 35, 40–48]. Furthermore, the rise in urinary CXCL10 has been demonstrated to precede the rise in serum creatinine [44, 48]. Urinary CXCL10 is sufficiently sensitive to detect underlying inflammation associated with both borderline and subclinical tubulitis as well as clinical rejection [25, 27, 28, 45, 48] and decreases after treatment of rejection [40, 43, 44, 46, 48]. Moreover, the persistence of elevated urinary CXCL10 has been associated with the early development of IFTA [49] and decreased renal allograft function at 6 months [48]. Elevated pre-transplant serum CXCL10 is also associated with decreased allograft survival [50, 51].

We evaluated the diagnostic performance of urinary CXCL10 in a prospective, unselected adult renal transplant population of 213 patients, and validated that urinary CXCL10 detected both subclinical inflammation (AUC 0.69, sensitivity 0.61 and specificity 0.72) and clinical rejection (AUC 0.74, sensitivity 0.63 and specificity 0.80) [25]. Several points should be noted from this analysis. First, subclinical rejection was only detected by surveillance biopsy. By definition, serum creatinine and proteinuria have a sensitivity and specificity approaching 0 for subclinical inflammation. Second, the urinary CXCL10 AUC was calculated in the entire cohort to determine true population-based diagnostic performance. Indeed, the subclinical inflammation group included borderline tubulitis (t1) and isolated vascular compartment inflammation; these milder phenotypes most likely would have decreased our ability to detect a difference. Third, when only highly selected patient groups were compared, as done in CTOT-04, urinary CXCL10 had a significantly inflated AUC 0.90 (sensitivity 0.90, specificity 0.82, p = 0.006) for distinguishing normal histology from clinical Banff ≥ IA rejection. Finally, using urinary CXCL10 to determine who should undergo biopsy would have spared two-thirds of unnecessary surveillance biopsies, while capturing significant subclinical inflammation in the remaining biopsies [25].

Taken together, these data suggest that urinary CXCL10 exceeds the current clinical standard (serum creatinine, proteinuria) for detecting alloimmune inflammation and that a chemokine-directed monitoring strategy could guide the rational use of surveillance biopsies. Urinary CXCL10 is also the only biomarker to be independently validated for detecting subclinical tubulitis in an unselected patient population [25, 27, 28], although CXCL9’s performance is very similar [27]. Furthermore urinary CXCL10 has been confirmed as a marker for subclinical tubulitis in pediatric renal transplant recipients [52], whereas CXCL9 has only been evaluated in a small subset of pediatric patients [26, 45]. Finally, urinary CXCL10 and CXCL9 are simple, cost-effective ELISA-based assays and therefore should be highly feasible for translating to clinical practice.

## Conclusions

Non-invasive renal allograft monitoring could play an important role in guiding the rational use of surveillance biopsies, assist in the titration of immunosuppression and help follow response to therapy. The advent of large, multicentre prospective observational cohort studies has significantly advanced biomarker development by allowing for the adequately powered evaluation of multiple biomarkers; this has narrowed an extensive list of novel markers identified in the “discovery” phase to a few key candidates. The utility of this cannot be understated, since biomarker discovery is frequently hampered with high false discovery rates; even if positive associations are demonstrated, it does not mean a biomarker will have a strong diagnostic performance.

So what lessons about biomarker development can be learned from these studies? It is clear that urinary CXCR3 chemokines can detect subclinical cellular rejection (outperform serum creatinine and proteinuria); rise prior to acute clinical rejection; decrease in response to therapy; have long-term prognostic significance; and these findings have been independently validated in several cohorts. Therefore, one question that arises is: what level of evidence should be considered acceptable for the evaluation of novel biomarker performance? Perhaps going forward, biomarker diagnostic performance should be evaluated in combination and against CXCR3 chemokines, instead of versus clinical measures alone.

While it might not be feasible, an ideal biomarker(s) would provide 100 % sensitivity with 100 % specificity. Therefore, another question that arises is how can biomarker discovery experiments be optimized to identify markers that improve on the overall performance of CXCR3 chemokines? All biomarker discovery research to date has been performed by simply comparing rejection versus control patients, with different variations on how these are defined. Instead of this general non-specific approach, perhaps targeted comparison of acute rejection patients with a false negative urinary CXCL9/CXCL10 reading should be compared to control patients. This may increase the potential yield for identifying additional combination marker(s) that improve on the overall diagnostic performance of CXCR3 chemokines alone.

Ultimately, it is critical that prospective, interventional trials be performed to determine if chemokine-based monitoring strategies improve clinically meaningful, long-term renal allograft outcomes for patients; and this last step is necessary to translate novel biomarkers from the bench-to-bedside. These data would help inform decision-making on the rational use of urinary chemokines and help determine what cut-offs should be utilized. Indeed, should a cut-off be set for higher sensitivity versus specificity? How much potential subclinical pathology is considered acceptable to miss? The risks, costs and workload of surveillance biopsies need to be balanced against the clinical consequences of untreated subclinical rejection, so more data is urgently needed. Therefore, prospective interventional chemokine-based monitoring trials should be undertaken to inform both the clinical utility and implementation of novel, non-invasive renal allograft surveillance.

### What are the key messages?

- Current non-invasive monitoring strategies are limited as serum creatinine cannot detect subclinical pathologies.
- Surveillance biopsies are not useful for frequent monitoring, due to their invasive nature.
- Ideally, a biomarker should be sensitive, specific and outperform current clinical monitoring standards.
- Robust validation studies in unselected, consecutive patient populations are essential to determine the true population-based diagnostic performance.
- Prospective interventional trials are urgently needed to evaluate the clinical efficacy of chemokine-based monitoring strategies.

## Abbreviations

AMR: Antibody-mediated rejection
AUC: Area under the curve receiver operating characteristic
BKV: Polyomva virus
CCL5: Chemokine (C-C motif) ligand 5, also known as RANTES
CCR1: C-C chemokine receptor type 1
CCR5: C-C chemokine receptor type 5
CD103: Cluster of differentiation 103
CD3ε: T-cell surface glycoprotein CD3 epsilon chain
CD4: Cluster of differentiation 4
CMV: Cytomegalovirus
CORR: Canadian Organ Replacement Registry
CTL: Cytotoxic T lymphocytes
CTOT: Clinical Trials in Organ Transplantation
CXCL9: C-X-C motif chemokine 10, also known as Mig
CXCL10: C-X-C motif chemokine 10, also known as IP10
CXCL11: C-X-C motif chemokine 11, also known as ITAC
CXCR3: Chemokine (C-X-C motif) receptor 3, also known as G protein-coupled receptor 9 (GPR9) and CD183
DSA: Donor specific antibody
ELISA: Enzyme-linked immunosorbent assay
FOXP3: Forkhead box P3
GN: Glomerulonephritis
IFTA: Interstitial fibrosis and tubular atrophy
IL8: Interleukin 8
mRNA: Messenger RNA
NPV: Negative predictive value
rRNA: Ribosomal RNA
qPCR: Quantitative polymerase chain reaction
SELDI TOF-MS: Surface enhanced laser desorption ionization time-of-flight mass spectrometry
TGFβ: Transforming growth factor β
Th1: T helper type 1
Tim3: T-cell immunoglobulin domain and mucin domain-3
USRDS: United States Renal Data System
UTI: Urinary tract infection
